# Preoperative medication use and postoperative delirium: a systematic review

**DOI:** 10.1186/s12877-017-0695-x

**Published:** 2017-12-29

**Authors:** Gizat M. Kassie, Tuan A. Nguyen, Lisa M. Kalisch Ellett, Nicole L. Pratt, Elizabeth E. Roughead

**Affiliations:** Quality Use of Medicines and Pharmacy Research Centre, School of Pharmacy and Medical Sciences, Sansom Institute for Health Research, University of South Australia, Adelaide, South Australia 5001 Australia

**Keywords:** Delirium, Elderly, Medication, Risk factor, Prevention, Medication related problem, Adverse drug event, Medication safety

## Abstract

**Background:**

Medications are frequently reported as both predisposing factors and inducers of delirium. This review evaluated the available evidence and determined the magnitude of risk of postoperative delirium associated with preoperative medication use.

**Methods:**

A systematic search in Medline and EMBASE was conducted using MeSH terms and keywords for postoperative delirium and medication. Studies which included patients 18 years and older who underwent major surgery were included. The methodological quality of included studies was assessed independently by two authors using the Newcastle-Ottawa quality assessment scale for cohort studies.

**Results:**

Twenty-nine studies; 25 prospective cohort, three retrospective cohort and one post hoc analysis of RCT data were included. Only four specifically aimed to assess medicines as an independent predictor of delirium, all other studies included medicines among a number of potential predictors of delirium. Of the studies specifically testing the association with a medication class, preoperative use of beta-blockers (OR = 2.06[1.18–3.60]) in vascular surgery and benzodiazepines RR 2.10 (1.23–3.59) prior to orthopedic surgery were significant. However, evidence is from single studies only. Where medicines were included as one possible factor among many, hypnotics had a similar risk estimate to the benzodiazepine study, with one significant and one non-significant result. Nifedipine use prior to cardiac surgery was found to be significantly associated with delirium. The non-specific grouping of psychoactive medication use preoperatively was generally higher with an associated two-to-seven-fold higher risk of postoperative delirium, while only two studies included narcotics without other agents, with one significant and one non-significant result.

**Conclusions:**

There was a limited number of high quality studies in the literature quantifying the direct association between preoperative medication use and postsurgical delirium. More studies are required to evaluate the association of specific preoperative medications on the risk of postoperative delirium so that comprehensive guidelines for medicine use prior to surgery can be developed to aid delirium prevention.

**Trial registration:**

This systematic review has been registered on PROSPERO International prospective register of systematic reviews (Registration number: CRD42016051245).

**Electronic supplementary material:**

The online version of this article (10.1186/s12877-017-0695-x) contains supplementary material, which is available to authorized users.

## Background

Delirium is a serious and common complication following major surgery; however, its pathophysiology is not clearly understood. The most important mechanism involves disturbances of the neurotransmitter system; comprising of insufficient acetylcholine activity, increase in dopamine and noradrenaline activities, and either an excess or shortage of serotonin and gamma-aminobutyric acid (GABA) activities [1]. The acetylcholine hypothesis has been supported by studies showing an increase in the occurrence of delirium in patients with higher level of serum anticholinergic activity [2].

A specific agent or medication class can induce delirium by affecting the actions of one or more of the above neurotransmitters. Medications with anticholinergic activity include antiparkinsonians (benztropine, trihexyphenidyl), narcotics (meperidine, codeine), first generation antihistamines (diphenhydramine, hydroxyzine), antispansmodics, antinauseants, histamine (H2)-receptor blockers (ranitidine), psychoactive medicines (tricyclic antidepressants, lithium), and cardiac medications (digoxin). Sedative-hypnotics such as benzodiazepines and fluoroquinolone antibiotics can precipitate delirium through their effect on GABA. Fluoroquinolone antibiotics also enhance glutamate activity. Dopaminergic antiparkinsonians and morphine may increase delirium risk by increasing dopaminergic actions [3, 4].

Studies have shown that the incidence of postoperative delirium ranges from 4.4% in patients undergoing cataract surgery in Israel [5] to 56% among elderly patients undergoing orthopaedic surgery in Sweden [6]. An Australian study reported that postoperative delirium is common even in people without known preoperative risk, affecting 28% of surgical patients admitted to surgical ICU [7].

A number of risk factors have been identified to independently predict postoperative delirium; these include older age, type of surgical procedure, smoking, alcohol use, comorbidities, medications, and preoperative cognitive and functional status [8]. Many of these factors are not modifiable, however, medications are potentially modifiable and are commonly implicated in delirium; associated with up to 39% of delirium cases in elderly medical and surgical patients [9]. High-risk medication use can occur preoperatively, perioperatively or postoperatively. While some peri and postoperative medication use may not be modifiable or avoidable, such as use of anaesthetics for surgical patients, preoperative medication use is potentially modifiable. The available evidence on the effect of perioperative and postoperative medication use on postoperative delirium has been reviewed [10, 11].

There have been no systematic reviews which have specifically described the association between preoperative medication use and postoperative delirium in patients undergoing major surgical procedures. The aim of this review was to evaluate the available evidence to determine the magnitude of risk and to identify the type of preoperative medicines associated with postoperative delirium. In addition, this review assessed the extent of association between the number of preoperative medications and delirium following surgical procedures.

## Methods

### Types of studies included in the review

A systematic literature search was undertaken for randomized controlled trials (RCTs), prospective cohort (PC), retrospective cohort (RC) and case-control studies that reported pre-operative medications as a risk factor for postoperative delirium. Studies that examined the effect of a medication by intentionally administering to patients for the purpose of treating or preventing delirium were not included in this review. Reviews, case series, case reports, conference abstracts and editorials were excluded. Studies were included if their participants were aged 18 years and over, undergoing either emergency or elective surgical procedures. We included studies published in English until August 2017 and used Diagnostic and Statistical Manual for Mental Disorders Criteria (DSM) or standardized tools for diagnosing delirium. The primary outcome of individual studies had to be either incidence, severity or duration of postoperative delirium or a combination of these events.

### Search strategy and identification of studies

Search strategies were developed for MEDLINE and EMBASE databases using relevant MeSH terms and keywords for two concepts: postoperative delirium and medication. The two concepts were combined and the result was limited to adults, English language and humans. The search strategies employed for both databases are shown on the Additional file 1. The reference list of relevant articles including systematic reviews on risk factors for delirium were searched manually to identify further studies.

Searching for studies was conducted by the primary investigator (GK) with the assistance of a health librarian and the co-authors. Endnote was used to manage and record identified search results. The titles and abstracts of individual studies were reviewed to select eligible studies for inclusion with the help of an abstract review form (Additional file 2). The review form extracted information including objectives of the study, study design, study setting, study population, study outcomes, delirium assessment tool used and availability of preoperative medication data from each article. Full articles were reviewed where it was not possible to decide inclusion based upon abstract review. This systematic review is reported according to the Preferred Reporting Items for Systematic Reviews and Meta-Analyses (PRISMA) statement guidelines [12] (Additional file 3).

### Data extraction and quality assessment

The primary investigator (GK) developed a data extraction form for eligible studies. Data from each article were collected. The methodological quality of included studies was assessed using the Newcastle-Ottawa quality assessment scale for cohort studies (NOS) [13]. According to this scale, a study can be awarded a maximum of nine stars; four stars for study selection, two stars for comparability and three stars for outcome sections. However, this scale does not provide a threshold number of stars for a study to be considered as good or poor quality. The quality of individual studies was assessed by the first author and checked by a co-author (TN) with discrepancy being resolved by discussion. One star was granted for the second item in the ‘outcome’ section where the study stated that patients were assessed for delirium for at least the first four postoperative days.

## Results

### Characteristics of included studies

**Fig. 1:**
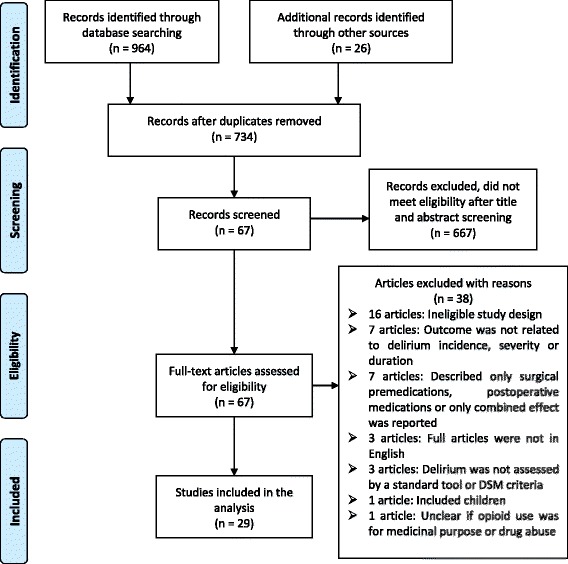
Flowchart of study selection and inclusion

The search yielded a total of 734 records after removing duplicates. We excluded 667 articles after title and abstract screening, leaving 67 articles for full article review. Twenty-nine of these articles met the inclusion criteria. The review process for selecting articles with reasons for exclusion is summarized in Fig. 1.

Twenty-five studies were prospective cohort studies, three studies were retrospective cohorts [14–16] and one was a post hoc analysis of RCT data [17]. Studies were conducted in USA (six studies), Canada (three studies), Netherlands (three studies), Sweden (two studies), Taiwan (two studies), Italy (two studies), Japan, Brazil, Switzerland, Norway, Australia, Thailand, France, Germany, Korea, Singapore and China (one study in each country).

Six different assessment methods were used to diagnose postoperative delirium. The confusion assessment method (CAM) was the most frequently used tool in 20 studies. The remaining studies used the memorial delirium assessment scale (MDAS) (*n* = 1 study), Neelon and Champagne confusion scale (NEECHAM) (*n* = 1 study), delirium symptom interview (DSI) (*n* = 1 study) and DSM-III or IV criteria (*n* = 6 studies) (Table 1). Three studies [18–20] excluded patients with dementia or Alzheimer’s disease. The CAM and Intensive Care Delirium Screening Checklist are the tools recommended by the Society of Critical Care Medicine (SCCM) as highly reliable for diagnosing delirium [21]. In this review, we included three studies in which delirium was diagnosed by three other tools; MDAS, NEECHAM Scale and DSI. Even though these three tools were not recommended as the most reliable tools by the SCCM, their reliability has been validated [22–24].

**Table 1:** Characteristics of studies included in the systematic review by type of surgery

Study (First Author, Publication year, Country)	Year of data collection	No of study participants	Age, Mean (SD)	Sex, Male (%)	Type of surgery	Diagnostic tool	Delirium incidence (%)	Study quality (star)^a^
Juliebo, 2009, Norway [35]	2005–2006	187	84.0^b^ (79–88)^c^	24	Orthopaedic	CAM	36	7
Kudoh, 2004, Japan [30]	NR	328	73.2	2	Orthopaedic	CAM	15	7
Muangpaisan, 2015, Thailand [43]	2010–2012	80	79.4 (7.9)	25	Hip fracture	CAM	45	7
Goldenberg, 2006, USA [25]	2000–2002	77	81.9 (7.5)	35	Hip fracture	CAM	48	7
Galanakis, 2001, Germany [18]	1998	105	74.9^b^ (60–98)^d^	29	Hip surgery	CAM	24	9
Duppils, 1999, Sweden [29]	1996–1997	225	85.4	33	Hip surgery	DSM-IV	20	6
Schuurmans, 2003, Netherlands [31]	1998–1999	92	82.7 (6.7)	13	Hip fracture	DSM-IV	20	7
Huang, 2017, Singapore [16]	2006	954	78^be^, 67 ^bf^	81	Total knee arthroplasty	DSM-IV	1	7
Mariscalco, 2012, Italy [36]	2004–2010	4659	67.8 (9.2)	79	Coronary revascularization	CAM-ICU	3	9
Afonso, 2010, USA [41]	2008	112	66 (19–84)^d^	57	Cardiac and thoracic aortic	CAM-ICU	34	7
Burkhart, 2010, Switzerland [17]	NR	113	74.3 (5.5)	68	Cardiopulmonary bypass	CAM	30	7
Santos, 2004, Brazil [38]	1996–1999	220	70.7	65	Coronary artery bypass graft	CAM-ICU	34	8
Rudolph, 2008, USA [39]	NR	42	68.1	83	Cardiac	CAM	29	8
Tan, 2008, USA [32]	2006	53	62.7	100	Cardiac	MDAS	23	6
Veliz-Reissmuller, 2007, Sweden [44]	NR	107	72.9 (5.4)	62	Elective cardiac	CAM	23	7
Katznelson, 2009b, Canada [34]	2006–2007	582	67.9	71	Elective and emergency vascular	NEECHAM	22	9
Van Der Mast, 1999, Netherlands [40]	NR	296	63.0 (11)	65	Elective cardiac	DSM-III-R	14	7
Katznelson, 2009c, Canada [37]	2005–2006	1059	63.6^g^	71	Cardiopulmonary bypass	CAM-ICU	12	9
Tully, 2010, Australia [27]	2007–2009	158	64.7 (10.6)	79	Coronary artery bypass graft	DSI	31	8
Benoit, 2005, Canada [8]	2000–2003	102	70.8 (8.2)	77	Elective abdominal aortic aneurysm	CAM	33	7
Tognoni, 2011, Italy [45]	NR	90	74.3 (0.40)	90	Urologic	CAM	9	7
Dai, 2000, Taiwan [26]	1995–1997	701	72.7 (6.3)	46	Elective orthopaedic and urologic	DSM-IV	5	7
Chen, 2015, Taiwan [14]	2009–2013	401	61.2 (11.2)	74	Hepatectomy for HCC	CAM	8	7
Jeong, 2016, Korea [15]	2014–2015	475	76.0^b^(65–96)^d^	45	Cancer surgery	DSM-IV	4	8
Brouquet, 2010, France [42]	2006–2008	118	81.3 (4.8)	48	Elective major abdominal	CAM	24	8
Behrends, 2013, USA [33]	2003–2011	472	38.1^h^	52	Major non-cardiac	CAM	29	7
Litaker, 2001, USA [28]	NR	500	67.0 (9.0)	NR	Elective major surgery	CAM	11	8
Xue, 2016, China [20]	2010–2015	358	75.1	NR	Transurethral resection of prostate	CAM	8	8
Brown, 2016, USA [19]	2012–2014	89	74^b^	53	Spine surgery	CAM/CAM-ICU	41	9

*NR* not reported, *SD* standard deviation, *DSI* delirium symptom interview, *DSM* diagnostic and statistical manual for mental disorders, *DOS* delirium observation screening, *MDAS* memorial delirium assessment scale, *NEECHAM* neelon and champagne confusion scale, *HCC* Hepatocellular carcinoma

^a^Study quality assessment as per NOS; ^b^Median; ^c^Interquartile range; ^d^Range; ^e^delirium group; ^f^non-delirium group; ^g^63.6% were > 60 years old; ^h^38.1% were > 75 years

Twelve studies described preoperative medication use in patients for cardiac surgery and eight investigated the effect of preoperative medication use in orthopaedic surgery. The incidence of postoperative delirium ranged from 1% in patients for total knee arthroplasty [16] to 48% in hip fracture patients (Table 1) [25].

Twenty-three studies assessed the relationship between some medication classes and postoperative delirium while nine studies investigated the effect of the total number of preoperative medicines on postoperative delirium. However, only four of the studies were designed to assess medicines as independent predictors of delirium. The remaining studies included medicines, either type or number, as a variable in predictive models.

### Study quality

The quality scores of studies ranged from six to nine stars with a median of seven stars. Twenty-six studies demonstrated that patients with preoperative medications were representative of the surgical patient population under study. These studies also indicated exposure to medications was ascertained by patient records, structured patient interview or by a combination of these methods. The absence of preoperative delirium in included patients was not sufficiently described or a separate analysis was not undertaken in two studies.

Five studies scored the maximum stars on ‘comparability’ with at least two important delirium risk factors controlled between the cohorts. Eleven of the 29 studies failed to get the maximum score in the ‘outcome’ section because either delirium was not assessed in a blinded manner; follow-up time was insufficient or results were subject to bias because of loss of patients to follow-up.

### Studies aimed to assess the association between medicine use and delirium

Only four of the 29 studies were conducted with the aim of assessing an association between specific preoperative medicine use and postoperative delirium. The association between postoperative delirium and preoperative use of beta-blockers (one study) and statins (three studies) was assessed in patients for cardiac surgery while preoperative benzodiazepines use (one study) was assessed in patients for orthopaedic surgery (Table 2).

**Table 2:** Studies designed to investigate the effect of preoperative use of specific medicines on postoperative delirium

Study (First author, publication year)	Preoperative medicines	Other covariates	OR or RR (95% CI)^c^	*P*-value
Katznelson, 2009b [34]	Beta-blockers	Age History of cerebrovascular accident Depression	OR 2.06 (1.18–3.60)	0.011
	Statins^a^		OR 0.56 (0.37–0.88)	0.011
Katznelson, 2009c [37]	Statins^a^	Older age Preoperative depression Preoperative renal dysfunction Complex cardiac surgery Perioperative intraaortic balloon pump support Massive blood transfusion	OR 0.54 (0.35–0.84)	0.010
Mariscalco, 2012 [36]	Statins^b^	Age group Cardiopulmonary bypass duration	OR 1.52 (0.97–2.37)	0.180
Kudoh, 2004 [30]	Benzodiazepines	Preoperative Mini-Mental State scores, anxiety scores, depression scores	RR 2.10 (1.23–3.59)	0.007

*CI* confidence interval, *OR* odds ratio, *RR* relative risk; ^a^ Protective effect; ^b^ Not associate; ^c^ only for preoperative medicines

### Predictor studies

Nineteen of the 29 studies assessed predictors of delirium risk where medicines were one of the risk factors assessed. The medications investigated included narcotics, antidepressants, other psychoactive medicines, anticholinergics, nifedipine and other cardiovascular agents (Table 3).

**Table 3:** Associations between preoperative medications and postoperative delirium reported in multivariate analyses of risk factor studies

Study (First author, publication year)	Preoperative medicines	Other covariates	Number of study participants	Number of patients with medications	OR or RR (95% CI)^e^	*P*-value
Chen, 2015 [14]	Hypnotics	Advanced age Prolonged operative time Decreased postoperative haemoglobin level	401	42	OR 3.07 (1.05–9.04)	0.041
Galanakis, 2001 [18]	Hypnotics and sedatives	Age Sex	105	21	OR 2.53 (0.79–8.04)	NS
Benoit, 2005 [8]	Psychoactive medicines	Lower education level Preoperative depression	102	0.18^a^ (0.4^c^), 0.5^b^ (0.8^c^)	OR 6.80^d^	0.005
Xue, 2016 [20]	Psychoactive medicines	Old age Pain intensity after surgery	358	64	OR 1.60 (0.65–3.93)	0.306
Duppils, 2000 [29]	Psychopharmacological drugs	Older age Cognitive impairment Pre-existing cerebrovascular disorders	225	88	OR 2.92 (1.33–6.39)	0.007
Dai, 2000 [26]	Narcotics and other psychoactive medicines	Age Pre-existing cognitive impairment	701	1.0^a^ (0.8^c^), 1.7^b^ (0.9^c^)	RR 6.56 (1.53–28.17)	< 0.05
Litaker, 2001 [28]	Narcotics	Previous delirium Age Pre-existing cognitive impairment	500	87	OR 2.70 (1.4–5.3)	Sig
Behrends, 2013 [33]	Narcotics	Age Sex History of central nervous system disorder Preoperative cognitive dysfunction Pain Blood transfusions	472	333	OR 1.02 (0.61–1.70)	0.940
Tully, 2010 [27]	Anticholinergic, selective serotonin re-uptake inhibitor and tricyclic drug	Major depression Aortic cross-clamp time Haemoglobin	158	12	OR 6.17 (1.27–30.12)	0.020
Tan, 2008 USA [32]	‘other’ anticholinergic medications	History of cerebrovascular disease High medical comorbidity Increased preoperative creatinine level Increased preoperative pain level	53	20	RR 2.31 (0.85–6.31)	0.100
Brown, 2016 [19]	Antidepressant	Lower baseline cognition Higher average baseline pain More intravenous fluid administered	89	26	OR 4.70 (1.03–21.5)	0.046
Benoit, 2005 [8]	Antihypertensive and antianginals	Lower education level Preoperative	102	0.8^a^ (0.8^c^), 0.4^b^ (0.5^c^)	OR 0.26^d^	0.016
Van Der Mast, 1999 [40]	Nifedipine	Old age Low level of albumin Poor physical condition High ratio of phenylalanine to the sum of isoleucine, leucine, valine, tyrosine, and tryptophan	296	53	OR 2.40 (1.0–5.8)	0.047
Jeong, 2016 [15]	DIM	Dementia Age Sex	475	200	OR 12.8(2.8–57.7)	< 0.001

*CI* confidence interval, *OR* odds ratio, *RR* relative risk, *Sig* reported as significant, *NS* reported as not significant, *DIM* medications whose adverse events, such as delirium, confusion or hallucination, were reported over 1% by the drug information database Micromedex®

^a^Average number of medicines in the non-delirium group; ^b^Average number of medicines in the delirium group; ^c^Standard deviation; ^d^CI not reported or data not sufficient to calculate; ^e^only for preoperative medicines

### Medication classes and agents studied

#### Psychoactive medications

Thirteen studies examined the effect of preoperative use of psychoactive medications on postoperative delirium in various groups of surgical patients [8, 14, 15, 18–20, 26–32]. Nine of these studies [8, 14, 15, 19, 26–30] reported a significant association between preoperative psychoactive medicine use and postoperative delirium with a two-to-seven-fold higher risk in patients who were taking these medicines prior to surgery. The risk of developing postoperative delirium in patients who were taking psychoactive medicines including narcotics preoperatively was reported to be more than six-times the risk of patients without these medicines in another study (RR = 6.56 [1.53–28.17]; *p* < 0.05) [26]. The study did not report stratified results for narcotics and other psychoactive agents separately. This association was not observed in one study among patients who underwent transurethral resection of prostate [20], in two small studies in patients after hip surgery [18, 31] and in another small study after cardiac surgery [32]. One study reported a thirteen times higher risk of developing delirium in patients who were on medicines inducing delirium prior to cancer surgery [15]. This study defined medicines inducing delirium as any medicine having a delirium-like adverse effect report of more than 1% according to the drug information database (Micromedex®).Most of the medications included in this list were centrally acting agents.

Four studies described the relationship between preoperative narcotic medication use and postoperative delirium [16, 26, 28, 33]. A high quality prospective cohort study showed that patients taking narcotic analgesics prior to admission for elective major surgery had a nearly three-fold (OR = 2.7 [1.37–5.3]) increased risk of developing postoperative delirium [28]. Patients who were taking opioids prior to total knee arthroplasty were also found at a higher risk of developing delirium following the procedure [16]. In patients for noncardiac surgery, there was no significant association between preoperative opioid use and postoperative delirium (OR = 1.02 [0.61–1.70]) [33].

#### Statins

Mixed results were reported by five studies which investigated the association between preoperative statin use and postoperative delirium [17, 34–37]. A prospective cohort study reported that the odds of delirium were reduced by 46% in patients who were using statins before cardiac surgery [37]. Another study conducted in the same hospital had similar findings in patients who underwent elective and emergency vascular surgery, where the risk of postoperative delirium was reduced by 44% in patients using statins prior to surgery [34]. This association was not observed in three studies: a post hoc analysis of data from a randomized controlled trial for assessing modifiable risk factors for postoperative delirium in elderly patients after elective cardiac surgery [17]; a prospective cohort study involving patients for hip fracture [35] and a large prospective observational study in patients for cardiac surgery [36].

#### Other cardiovascular agents

Seven studies analysed the effect of preoperative use of antihypertensive and cardiovascular medications including diuretics, calcium channel blockers, beta-blockers, ACE inhibitors and nitrates on postoperative delirium [8, 19, 34, 38–41]. Preoperative use of these medications was found to be independently associated with postoperative delirium in three of these studies [8, 34, 40]. The first study [40] reported that preoperative use of nifedipine was associated with a more than two-fold increased risk of developing postoperative delirium in patients who underwent cardiac surgery (OR = 2.40 [1.0–5.8]). The second study found a protective effect of antihypertensive and antianginal medications for postoperative delirium (OR = 0.26) [8]. The third [34] reported an increased risk (OR = 2.06[1.18–3.60]) of delirium following elective and emergency vascular surgery in patients who used beta-blockers including acebutalol, atenolol, bisoprolol, carvedilol and metoprolol preoperatively. In the remaining four risk factor studies antihypertensives were not found to be strong predictors to be included in the multivariate models.

### Number of preoperative medicines

Nine studies [18, 25, 31, 35, 39, 42–45] included the total number of preoperative medicines in their predictive models for postoperative delirium. Only one study [25] found it to be an independent predictor of postoperative delirium. In this study, patients who were taking more than three medications preoperatively had a higher risk of developing delirium after hip fracture surgery than patients who were taking less than three medications prior to the procedure (OR = 33.6 [1.9–591.6]) [25].

## Discussion

Determining the magnitude of delirium risk associated with the number and type of preoperative medicines is critical in designing interventions to reduce the use of these medications prior to surgery to decrease the risk of postoperative delirium. This systematic review has shown that there were only four studies that specifically assessed the association between preoperative medicine use and postoperative delirium. In majority of the studies, medicines were included as one of several potential risk factors for postoperative delirium. These studies are usually underpowered as the number of patients who were on the medications of interest was not sufficient to detect the delirium risk difference. Collectively the studies show that psychoactive medicines, antidepressants, beta-blockers and nifedipine were found to be a predictor of delirium; however, several limitations were apparent.

The main limitation of studies assessing psychoactive medicine use was the failure to report the indications for the medications investigated and not adjusting for comorbidities. This made it difficult to determine whether the higher risks of delirium reported were due to the medicines themselves or the health conditions for which the medicines were prescribed. In addition, some of the studies [14, 26, 27] analysed psychoactive medicines as a group without specifying individual medications because of the small number of patients taking medications in each class. Despite these limitations, overall evidence shows that preoperative use of psychoactive medications was associated with a two-to seven-fold increased risk of postoperative delirium, including a study that excluded patients with current psychosis or those on antipsychotics [27]. A prospective observational study involving 89 patients for spine surgery showed that preoperative antidepressant use has been associated independently with postoperative delirium [19]. This study did not also specify the types of antidepressants investigated.

The four studies [18, 20, 28, 32] which showed no association between psychoactive medicine use and postoperative delirium had some sources of bias. Their main limitation was the small number of patients involved in these studies reducing the power of individual studies. A study that had the largest sample size from this group [20], excluded patients with preoperative depression or cognitive impairment and dementia. So, patients who were more likely to be taking psychoactive medications had a higher risk of exclusion from the study which underestimated the possible risk related to these medications. While there was a study in this review [18] that included depressed patients, its power was limited by the small sample size that made conclusions difficult. The other two studies [31, 32] which involved 92 and 53 patients respectively did not specify the types of psychoactive medications assessed.

One prospective cohort study showed that preoperative narcotic medication use was associated with a nearly three-fold increase in delirium risk following elective major surgery [28]. Narcotics could contribute to risk of delirium through their anticholinergic, dopaminergic or their sedative effects. Another study which reported a more than six-fold increase in the risk of postoperative delirium among patients undergoing orthopaedic or urologic surgery analysed preoperative narcotic use collectively with other psychoactive medications [26], which makes it not possible to determine the risk contributed by preoperative narcotic analgesics.

The association between preoperative narcotic use and postoperative delirium was not found in patients undergoing noncardiac surgery [33]. This study was less reliable as only delirium cases occurring in the first postoperative day were considered excluding delirium cases developing subsequent to day one. The effect of chronic narcotic use on the onset of delirium on postoperative days other than the first day was not assessed.

Use of statins preoperatively was reported to either decrease risk [34, 37] or to have no association with the risk of postoperative delirium [17, 35, 36]. The reported protective effect of statins may be considered as plausible due to their anti-inflammatory action [46], however, this was not supported by an earlier retrospective study [47] that showed an increased risk of postoperative delirium with the use of statins prior to surgery. Both studies which reported a lower risk of delirium with preoperative statin use were conducted in the same hospital and in patients who underwent cardiac surgery. Therefore, further sufficiently powered prospective studies are required to be conducted in other settings and in patients having other types of surgery to be conclusive.

An increased risk of postoperative delirium was reported in patients having elective and emergency vascular surgery who used beta-blockers including acebutalol, atenolol, bisoprolol, carvedilol and metoprolol preoperatively [34]. Beta-blockers are associated with a number of central nervous system (CNS) side effects including confusion, hallucinations, nightmare, insomnia and sleep disturbance [48] by affecting cerebral levels of serotonin and melatonin [49]. Abnormalities in sleep including insomnia are one of the risk factors for delirium. Earlier evidence shows that the CNS effects differ across beta-blockers [50]. However, the risk associated with each beta-blockers investigated in this study was not reported.

Another study reported that vasoactive medications, defined as antihypertensive and antianginal agents, had a protective effect on postoperative delirium [8]. However, this study did not indicate the specific types of antihypertensive and antianginal agents investigated. Nifedipine which is an antihypertensive and antianginal drug, was reported to have a significant association (OR = 2.40 [1.0–5.8]) with postoperative delirium after cardiac surgery [40]. This effect could be secondary to the potential reflex tachycardia by nifedipine, the most potent dihydropyridine, especially when short-acting formulations are used [51]. The study did not indicate the type of nifedipine formulation used by study participants to enable interpretation of the finding further from this perspective.

Generally, it was difficult to assess whether the risk of delirium associated with a certain medication (class), for instance opioids, differs by surgery type. This is due to the presence of other confounders and the differences in study designs employed in each study included in this review. Moreover, some of the medications, for example nifedipine, were investigated only in one study.

Only one of the nine studies [25] that considered the number of preoperative medicines as a risk variable reported a significant association. However, the small number of patients (77 patients) involved in this study limits the generalizability of the significant finding. In addition, the very wide confidence interval (OR = 33.6 [1.9–591.6]) raises concern about the precision of the estimate. It was not possible to compare findings about the relationship between the total number of preoperative medications and postoperative delirium across studies in this review. This is because of significant differences in the definitions for polypharmacy employed in each study, types of surgery and patient groups included.

Another limitation was the use of categorical data (e.g. three or more, five or more medicines) rather than continuous data which may have obscured the effects. Preoperative use of more than three medications was found to be significantly associated with delirium following hip fracture surgery in one study [25]. However, this association was not observed in two studies conducted in patients for hip surgery even if a higher medication number was set as a cut-off point in categorizing patients [18, 35]. This lack of association could be because patients taking several medicines were living with multiple comorbidities that required medications and these might have contributed to delirium more than polypharmacy did.

This systematic review has some additional limitations. We were unable to conduct meta-analyses to have the pooled risk for medications associated with delirium due to the heterogeneity of studies in terms of patient population, surgery type and medications investigated. Some studies which could fulfil our inclusion criteria might not be included in the review if published in languages other than English. Moreover, literature for this review was searched from two databases, Medline and EMBASE. Potential studies for this review from sources not indexed to one of these databases might be missed.

## Conclusions

Findings from studies included in this review indicate the presence of association between postoperative delirium and use of antidepressants, other psychoactive medicines, beta-blockers and nifedipine prior to surgery.

Evidence from prospective cohort studies showed that patients who were taking psychoactive medications preoperatively had a higher risk of developing delirium after surgery than patients who were not using these medications. However, provision of specific recommendations on particular medicines is difficult as most studies analysed and collectively reported a range of CNS acting medicines under psychoactive medications.

Overall evidence suggests that preoperative use of statins did not reduce the risk for postoperative delirium following cardiac surgery. Preoperative use of beta-blockers and nifedipine was reported to increase risk of postoperative delirium by more than two-fold. Studies that included the number of preoperative medicines a patient taking as one of the potential variables did not generally show significant relationship with postoperative delirium.

All the studies included in this review are observational studies or a post hoc RCT. None of the studies evaluated the effect of medications on the severity or duration of delirium as there could be a possibility that medicines affect these outcomes. Studies included in the review, except one study, did not indicate if delirium risk was related to dosage, nor did they attempt to link the increased risk with the indications for the medication investigated. More methodologically rigorous and adequately powered studies are required to evaluate the effects of specific preoperative medications on incidence, duration and severity of postoperative delirium.

## Additional files

Additional file 1: Search strategies and results (DOCX 18 kb)

Additional file 2: Title and Abstract Review Forms (DOCX 43 kb)

Additional file 3: PRISMA Checklist (DOC 64 kb)

## Abbreviations

CAM: Confusion assessment method
CNS: Central nervous system
DSI: Delirium symptom interview
DSM: Diagnostic and statistical manual for mental disorders criteria
GABA: Gamma-aminobutyric acid
ICU: Intensive Care Unit
MDAS: Memorial delirium assessment scale
NEECHAM: Neelon and champagne confusion scale
PC: Prospective cohort
PRISMA: Preferred reporting items for systematic reviews and meta-analyses
RC: Retrospective cohort
RCT: Randomized controlled trial
SCCM: Society of critical care medicine
