# Supplementary material for: Preoperative medication use and postoperative delirium: a systematic review
Source: BMC Geriatr. 2017 Dec 29;17:298. doi: 10.1186/s12877-017-0695-x (PMC5747155; doi:10.1186/s12877-017-0695-x)
Supplement: Supplementary file 1 — Search strategies and results (DOCX 18 kb) [file 12877_2017_695_MOESM1_ESM.docx]

**Additional file 1.** Search strategies and results

1. **MEDLINE (Search date: 31/08/2017)**

| **Search** | **Result** |
| --- | --- |
| 1. exp Confusion/ | 11967 |
| 1. (deliri* or confus* or acute confusion* or acute mental status change* or altered mental status* or organic brain syndrome or acute brain failure).mp. [mp=title, abstract, original title, name of substance word, subject heading word, keyword heading word, protocol supplementary concept word, rare disease supplementary concept word, unique identifier] | 69295 |
| 1. Postoperative Complications/ | 333509 |
| 1. 1 or 2 | 69295 |
| 1. 3 and 4 | 2061 |
| 1. ((post?operat* or postoperat* or operation* or operated or surgical or surgery) adj3 (deliri* or acute confusion* or acute mental status change* or altered mental status* or organic brain syndrome or acute brain failure)).mp. [mp=title, abstract, original title, name of substance word, subject heading word, keyword heading word, protocol supplementary concept word, rare disease supplementary concept word, unique identifier] | 1271 |
| 1. 5 or 6 | 2600 |
| 1. exp therapeutic uses/ | 4961019 |
| 1. exp nonprescription drugs/ or exp prescription drugs/ | 10092 |
| 1. exp polypharmacy/ | 3714 |
| 1. (medication* or pharmaceutical* or pharmacol* or prescription* or drug* or medicin* or Prescription drug or nonprescription drug or prescrib* drug or prescrib* medicin* or prescrib* medication*).mp. [mp=title, abstract, original title, name of substance word, subject heading word, keyword heading word, protocol supplementary concept word, rare disease supplementary concept word, unique identifier] | 3778498 |
| 1. 8 or 9 or 10 or 11 | 6955368 |
| 1. 7 and 12 | 836 |
| 1. limit 13 to (english language and humans and "all adult (19 plus years)") | 441 |

1. **EMBASE (Search date: 31/08/2017)**

| **Search** | **Result** |
| --- | --- |
| 1. exp Confusion/ | 27027 |
| 1. (deliri* or confus* or acute confusion* or acute mental status change* or altered mental status* or organic brain syndrome or acute brain failure).mp. [mp=title, abstract, heading word, drug trade name, original title, device manufacturer, drug manufacturer, device trade name, keyword] | 128002 |
| 1. Postoperative Complications/ | 59483 |
| 1. 1 or 2 | 128002 |
| 1. 3 and 4 | 281 |
| 1. ((post?operat* or postoperat* or operation* or operated or surgical or surgery) adj3 (deliri* or acute confusion* or acute mental status change* or altered mental status* or organic brain syndrome or acute brain failure)).mp. [mp=title, abstract, heading word, drug trade name, original title, device manufacturer, drug manufacturer, device trade name, keyword] | 2357 |
| 1. 5 or 6 | 2558 |
| 1. exp therapeutic uses/ | 2323767 |
| 1. exp nonprescription drugs/ or exp prescription drugs/ | 18540 |
| 1. exp polypharmacy/ | 11717 |
| 1. (medication* or pharmaceutical* or pharmacol* or prescription* or drug* or medicin* or Prescription drug or nonprescription drug or prescrib* drug or prescrib* medicin* or prescrib* medication*).mp. [mp=title, abstract, heading word, drug trade name, original title, device manufacturer, drug manufacturer, device trade name, keyword] | 11746869 |
| 1. 8 or 9 or 10 or 11 | 12063145 |
| 1. 7 and 12 | 1143 |
| 1. limit 13 to (human and english language and (adult <18 to 64 years> or aged <65+ years>)) | 523 |
