# Supplementary material for: Preoperative medication use and postoperative delirium: a systematic review
Source: BMC Geriatr. 2017 Dec 29;17:298. doi: 10.1186/s12877-017-0695-x (PMC5747155; doi:10.1186/s12877-017-0695-x)
Supplement: Supplementary file 2 — Title and Abstract Review Forms (DOCX 43 kb) [file 12877_2017_695_MOESM2_ESM.docx]

**Additional file 2:** Title and Abstract Review Forms

**Title Review Form**

Does this article **POTENTIALLY** apply to the following objective?

**Objective:** To review/assess the effect of patients’ prior medication use on postoperative delirium in the elderly

**Abstract Review Form**

| **Inclusion/Exclusion Criteria** |
| --- |
| **Study subjects:** Human beings  **Yes No Unclear**  **Language:** Is study published in English?  **Yes No Unclear** |
| **Study design:** Is the study design one of the following?  Randomized control trial  Quasi-experimental  Prospective cohort  Retrospective cohort  Case-control  (Exclude reviews, study protocols, case series, case reports, editorials, commentaries, letters to editor, Conference papers)  **Yes No Unclear** |
| **Study Setting:**  Is the study conducted in a healthcare setting (e.g. in a hospital)?  **Yes No Unclear**  Does this study include patients in the perioperative period?  **Yes No Unclear** |
| **Study Participants:**  Are participants of this study aged 18 years or older?  **Yes No Unclear** |
| **Delirium assessment tool:** Has the study used a validated assessment tool for diagnosis of delirium? (One or more of DSM, CAM, DRS, CAM–ICU, DSI, ICDSC, DOS, and MDAS)?  **Yes No Unclear** |
| **Study outcome:** Does outcome of the study include any of the following?   - Incidence of delirium - Severity of delirium - Duration of delirium   **Yes No Unclear** |
| **Medication history:** Were data on patient medication use prior to surgery collected and/or reported?  **Yes No Unclear** |

**DSM**, Diagnostic and Statistical Manual of Mental Disorders (American Psychiatric Association, Arlington, VA); **CAM**, Confusion Assessment Method; **CAM–ICU**, Confusion Assessment Method–Intensive Care Unit; **DRS**, Delirium Rating Scale; **DSI**, Delirium Symptom Interview; **ICDSC**, Intensive Care Delirium Screening Checklist; **MDAS**, Memorial Delirium Assessment Scale.
